# Interleukin 7-expressing fibroblasts promote breast cancer growth through sustenance of tumor cell stemness

**DOI:** 10.1080/2162402X.2017.1414129

**Published:** 2018-01-03

**Authors:** Maximilian Boesch, Lucas Onder, Hung-Wei Cheng, Mario Novkovic, Urs Mörbe, Sieghart Sopper, Guenther Gastl, Wolfram Jochum, Thomas Ruhstaller, Michael Knauer, Burkhard Ludewig

**Affiliations:** a Institute of Immunobiology, Kantonsspital St. Gallen, Rorschacherstrasse 95, St. Gallen, Switzerland; b Internal Medicine V, Medical University of Innsbruck, Anichstrasse 35, Innsbruck, Austria; c Institute of Pathology, Kantonsspital St. Gallen, Rorschacherstrasse 95, St. Gallen, Switzerland; d Breast Center, Kantonsspital St. Gallen, Rorschacherstrasse 95, St. Gallen, Switzerland

**Keywords:** Breast cancer, tumor microenvironment, interleukin 7, cancer-associated fibroblast, cancer stem cells, Cxcl12

## Abstract

The tumor microenvironment harbors cancer-associated fibroblasts that function as major modulators of cancer progression. Here, we assessed to which extent distinct cancer-associated fibroblast subsets impact mammary carcinoma growth and cancer cell stemness in an orthotopic murine model. We found that fibroblasts expressing the Cre recombinase under the control of the interleukin 7 promoter occupied mainly the tumor margin where they physically interacted with tumor cells. Intratumoral ablation of interleukin 7-expressing fibroblasts impaired breast tumor growth and reduced the clonogenic potential of cancer cells. Moreover, cDNA expression profiling revealed a distinct oncogenic signature of interleukin 7-producing fibroblasts. In particular, *Cxcl12* expression was strongly enhanced in interleukin 7-producing fibroblasts and cell type-specific genetic ablation and systemic pharmacological inhibition revealed that the CXCL12/CXCR4 axis impacts breast tumor cell stemness. Elevated expression of *CXCL12* and other stem cell factors in primary human breast cancer-associated fibroblasts indicates that certain fibroblast populations support tumor cell stemness and thereby promote breast cancer growth.

## Introduction

Breast cancer (BC) is the most frequent tumor type in women^1^ and clinically presents as a heterogeneous disease. Several molecular and histological subtypes can be distinguished, which differ in clinical behavior and therefore require tailored therapy, such as HER2-positive, hormone receptor-positive (‘luminal’) and triple-negative (‘basal’) disease.^2^ BC can disseminate during the early phases of the disease,^3^^,^^4^ which predisposes to distant metastasis formation and life-threatening systemic disease. BC was the first solid tumor to demonstrate a causal role of cancer stem cells (CSCs) in tumor growth and disease pathogenesis.^5^ Breast CSCs mediate metastasis and function as a primary source of relapse, owing to their inherent propensity for quiescence and drug resistance.^6^^,^^7^ Apart from cell-intrinsic factors that mediate their persistence under treatment,^8^ CSCs are also characterized by marked phenotypic and functional heterogeneity that probably compromises the success of current therapies.^9^ Hence it is reasonable to assume that CSCs require particular supporting niches to retain stemness.^10^^,^^11^ Targeting the CSC niche in the tumor microenvironment (TME) therefore represents an attractive therapeutic concept to reduce metastasis and re-growth after clinical remission.

Tumor cells grow in highly variable environments that are formed by complex and dynamic networks of non-transformed cells including immune cells, fibroblasts, and endothelial cells. The human breast TME is particularly rich in fibroblastic stromal cells, while immune and endothelial cells are relatively sparse.^12^ The lack of prominent immune cell infiltration or highly active angiogenesis renders BC generally less eligible for checkpoint blockade and anti-angiogenesis, respectively. Hence, targeting of the fibroblastic tumor stroma, even though less advanced in its clinical development, is promising. Indeed, fibroblastic stromal cells engage with BC cells to promote metastasis *via* CCL5^13^ and cancer-associated fibroblast (CAF)-derived signals select for BC cells with high bone-metastatic potential.^14^ CAFs also promote the stemness of BC cells through a CCL2/NOTCH1 axis,^15^ and POSTN expressed in the fibroblastic tumor stroma is necessary for breast CSC maintenance.^16^ However, two recent studies have revealed a tumor-suppressive role of stromal elements and CAFs in cancer,^17^^,^^18^ a finding that highlights the plasticity and heterogeneity of fibroblasts in malignant diseases. It is therefore important to assess whether different subsets of CAFs exist in BC and to determine whether particular CAF subsets promote stemness of mammary carcinoma cells.

Here, we utilized bacterial artificial chromosome (BAC)-transgenic mice to track and restrain interleukin 7 (*Il7*)-expressing CAFs in a model of orthotopic BC. We found that *Il7*-expressing CAFs promoted breast tumor growth and provided critical niches for maintenance of BC stemness. CXCL12 was identified as an important niche factor in *Il7*-expressing CAFs, indicating that the CXCL12/CXCR4 pathway may serve as a therapeutic anti-CSC target.

## Results

### Interaction of Il7-expressing fibroblasts with breast cancer cells

**Figure 1.**
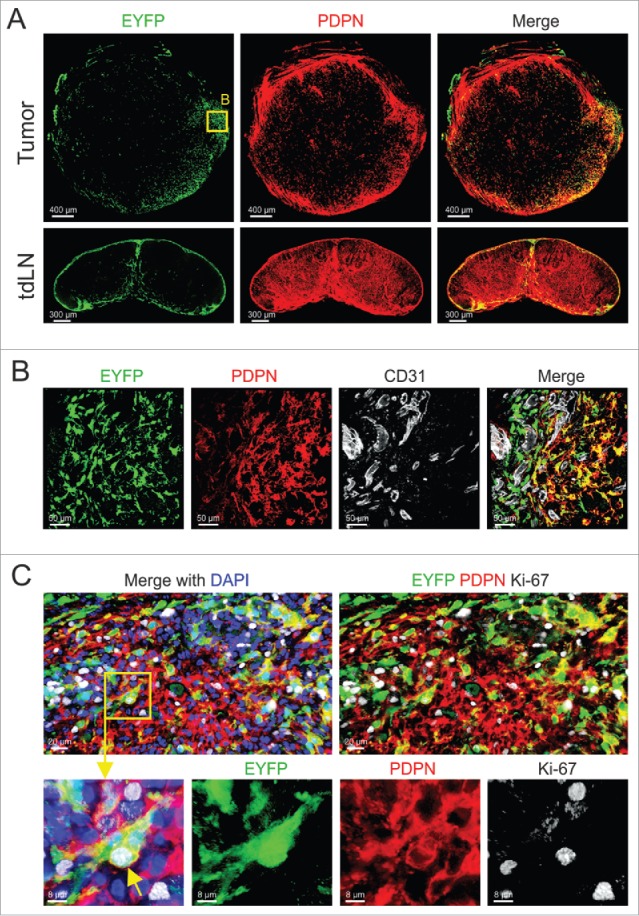
Characterizing *Il7*-expressing fibroblasts in syngeneic orthotopic breast tumors. (A) 5 × 10^5^ E0771 cells were grafted in the MFP of Il7-EYFP mice, and tumors and tdLNs were harvested at day 14. Tissue sections were stained with the indicated antibodies and analyzed by confocal microscopy. (B) Boxed area close-up of (A). (C) Confocal microscopic analysis of PDPN-expressing EYFP^+^ CAFs for Ki-67 expression. Data are representative examples of at least three independently performed experiments (n > 3).

Podoplanin (PDPN) expression contributes to the phenotypic identity and function of fibroblasts, both in secondary lymphoid organs^19^ and in various cancers.^20^ Here, we first assessed whether orthotopically growing E0771 murine mammary carcinomas harbor PDPN^+^ fibroblasts and found that PDPN expression was mainly restricted to cells located at the tumor margin (Fig. 1A). Since PDPN-expressing fibroblasts in secondary lymphoid organs are an abundant source of IL7 and thereby regulate T cell homeostasis,^21^ we determined the abundance and location of *Il7*-expressing fibroblasts in E0771 tumors. To this end, we utilized mice that express the Cre recombinase under the control of the *Il7* promoter^22^ and crossed them to the R26R-EYFP reporter strain^23^ (Il7-Cre R26R-EYFP mice, here referred to as Il7-EYFP mice). We found that E0771 tumors grown in Il7-EYFP hosts harbored transgene-expressing cells in regions with high abundance of PDPN^+^ cells (Fig. 1A). Consistent with previous data,^22^ EYFP^+^ cells were also detected in the subcapsular sinus and the medulla of tumor-draining lymph nodes (tdLNs) (Fig.1A). High resolution microscopic analysis revealed a large fraction of *Il7*-expressing CAFs in the vicinity of CD31^+^ blood vessels (Fig.1B and Supplementary Fig. 1A-C). Moreover, the cells were PDPN-positive, but lacked the expression of the myofibroblast marker alpha smooth muscle actin (αSMA) (Fig. 1B and data not shown). Ki-67 staining demonstrated *in situ* proliferative potential of *Il7*-expressing PDPN^+^ CAFs (Fig. 1C) suggesting that the cells are engaged in dynamic cellular interactions in the tumor margin.

**Figure 2.**
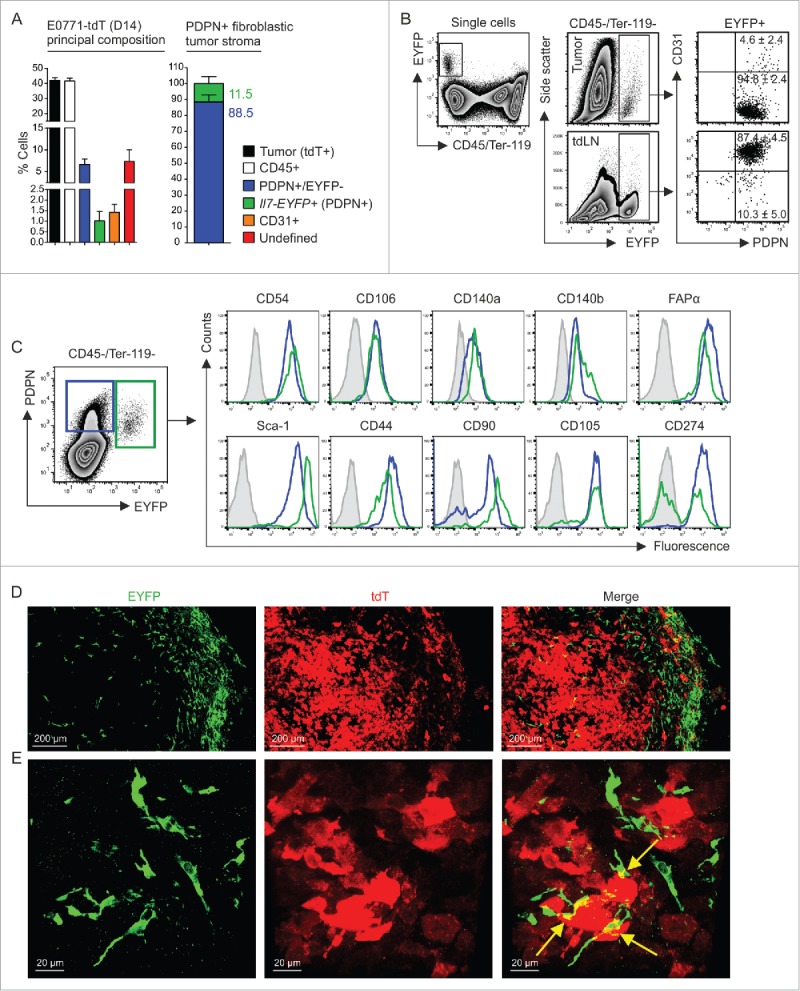
Cellular composition of mammary carcinomas in Il7-EYFP mice. (A) Flow cytometry-based enumeration of the indicated cell populations at day 14 post inoculation of 5 × 10^5^ E0771-tdT cells (n = 6–10). Note that EYFP^+^ cells are PDPN^+^. (B) Flow cytometric characterization of EYFP^+^ cells from tumors and tdLNs (n = 4–6). (C) Comparative flow cytometric profiling of EYFP^+^ and EYFP^−^PDPN^+^ breast CAFs (n = 6–8). (D+E) Confocal microscopic analysis of EYFP^+^ and EYFP^−^PDPN^+^ breast CAFs and tdT-labeled tumor cells (representative images from four independently performed experiments).

To better define the phenotype and properties of E0771 mammary carcinoma cells, we fluorescently labeled the cells by introducing the tdTomato (tdT) gene. Labeling of the cells with the red fluorescent marker facilitated discrimination of host cell types and hence allowed for quantification of the cellular composition of the tumor. As expected, cancer cells and CD45^+^ leukocytes formed the main cell populations with >40% of all nucleated cells, while EYFP^−^PDPN^+^ and EYPF^+^ stromal cells formed only minor populations (Fig. 2A, left panel). Notably, Il7-expressing CAFs accounted for roughly 10% of the fibroblastic tumor stroma (Fig. 2A, right panel). While the Il7-Cre transgene targets mainly PDPN^+^CD31^+^ lymphatic endothelial cells in lymph nodes (Fig. 2B, lower panels), the majority of EYFP^+^ cells in E0771 tumors were non-endothelial CAFs that expressed PDPN (Fig.2B, upper panels). Moreover, EYFP^+^ CAFs shared a set of mesenchymal stromal cell markers (e.g., CD54/ICAM-1, CD106/VCAM-1, CD140 a/PDGFRα, CD140b/PDGFRβ and FAPα) with their EYFP^−^PDPN^+^ counterparts (Fig.2C). Confocal microscopic analysis of tdT-positive E0771 tumor confirmed the multi-layered arrangement of EYFP^+^ fibroblastic stromal cells at the outer limit of the tumor parenchyma (Fig.2D) and revealed direct physical connections between the two cell types (Fig.2E). To establish broader relevance for *Il7*-producing breast CAFs, we transplanted 4T1 tumors to F1 (Il7-EYFP (C57BL/6; BALB/c) mice (Supplementary Fig. 2A and B). We found that EYFP^+^ cells in 4T1 tumors cells occurred in similar frequencies and displayed a comparable phenotype (Supplementary Fig. 2C) when compared to EYFP^+^ cells in E0771 tumors. Moreover, confocal microscopic analysis showed a similar morphology and localization of EYFP^+^ in 4T1 tumors including the spatial association with CD31^+^ blood vessels in the tumor margin (Supplementary Fig. 2 D and E). These data suggested that *Il7*-producing CAFs may contribute to the progression of tumor growth.

### Ablation of Il7-producing CAFs impairs breast tumor growth and tumor cell stemness

**Figure 3.**
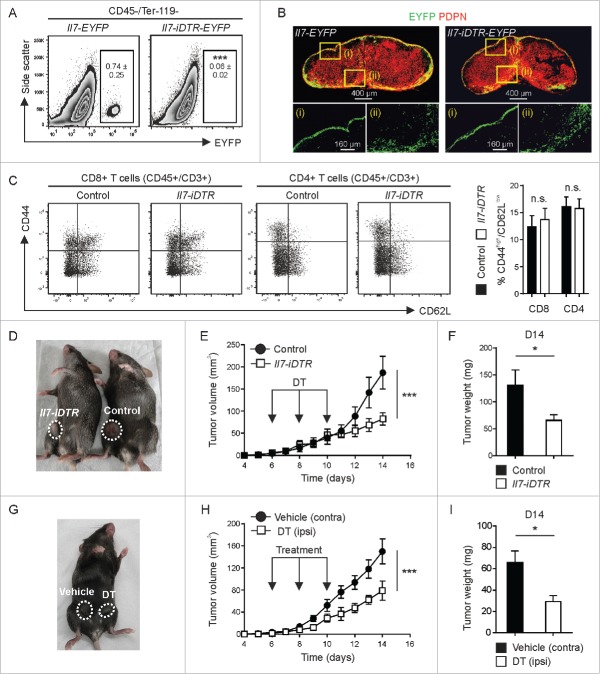
Impact of toxin-mediated ablation of *Il7*-expressing fibroblasts on breast tumor growth. E0771 cells were orthotopically grafted in Il7-iDTR/EYFP mice and transgene-expressing CAFs were ablated by intratumoral administration of DT (1 ng on days 6, 8 and 10). (A) Flow cytometric assessment of cell ablation in day 14 tumors (n = 4–10). (B) Assessment of the integrity of the fibroblastic reticular cell network in tdLNs using confocal microscopy. (C) Flow cytometric analysis of T cell activation in the tdLN (n = 5). (D-F) Unilateral tumors were induced in Il7-iDTR or Cre-negative control mice (iDTR-positive genetic background), and susceptible CAFs were ablated using treatment with DT, as indicated. Tumor growth was monitored on a daily basis and tumor weight was recorded on day 14 (n = 8–9). (G-I) Bilateral tumors were induced in Il7-iDTR mice and only the ipsilateral side was treated with DT, while the contralateral side was treated with vehicle (PBS) (n = 8). Microscopy data are representative examples of two independently performed experiments. Statistical testing used: Student's t-test for panels A, C, F and I, and repeated measures ANOVA for panels E and H (*p < 0.05, ***p < 0.001).

To dissect whether and to what extent *Il7*-expressing CAFs impact the growth of BC cells, we crossed Il7-Cre mice with the R26R-iDTR^24^ strain to render a distinct CAF subset susceptible to diphtheria toxin (DT)-mediated ablation. Using intratumoral injection of low doses of DT, we achieved ablation of *Il7*-expressing CAFs (Fig.3A), while the infrastructure of the tdLNs remained intact (Fig.3B). Integrity of the tdLN was further assessed by flow cytometry (Supplementary Fig. 3A and B) revealing unperturbed T cell homeostasis in the tdLN (Fig. 3C). This therapeutic intervention substantially inhibited tumor growth (Fig.3D-F) indicating that *Il7*-expressing CAFs are important for BC propagation. Next, we used bilateral injection of tumor cells and treatment with DT vs. vehicle in the same mouse to rule out off-target effects of the compound. Again, tumor growth was significantly reduced when *Il7*-expressing CAFs were ablated (Fig. 3G-I) indicating that a subset of tumor fibroblasts can regulate the growth of mammary carcinoma cells.

**Figure 4.**
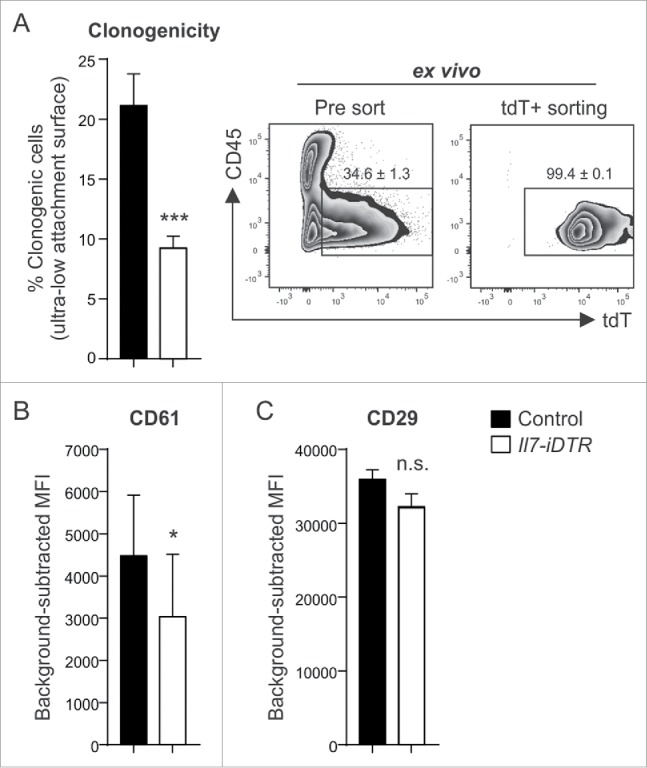
Impact of ablation of *Il7*-expressing fibroblasts on CSC properties. E0771-tdT cells were orthotopically grafted in Il7-iDTR or Cre-negative control mice, and ablated and non-ablated tumors were analyzed for phenotypic and functional CSC markers at day 14. (A) E0771-tdT cells were sorted and the clonogenic potential of the recovered cells was investigated under ultra-low attachment conditions (left panel) (n = 9–10). Post sort purity of the input cells routinely exceeded 98% (right panel). (B+C) Flow cytometric assessment of breast CSC marker expression by E0771-tdT cells recovered from ablated and non-ablated tumors, expressed as background-subtracted MFI (n = 9–10). Statistical testing used: Student's t-test for panels A-C (*p < 0.05, ***p<0.001).

Since *Il7*-expressing stromal cells in secondary lymphoid organs contribute to the regulation of immune cell function,^21^^,^^22^ we assessed first whether the local ablation of these cells in the tumor would affect immune cell recruitment. We found that DT-treated tumors harbor similar numbers of CD45^+^ leukocytes, T cells and myeloid cell subsets one day after the last treatment (Supplementary Fig. 4 A-C). Likewise, RT-PCR analysis revealed no significant changes in the expression of inflammatory mediators such as *Il6, Ccl2* and *Tnf* (Supplementary Fig. 4D-F). Considering that BC development and growth is determined by stemness properties of tumor cell subsets,^9^^,^^10^ we assessed various CSC properties of *ex vivo*-purified E0771-tdT cells. Cancer cells isolated from ablated tumors showed a markedly reduced clonogenic potential under stem cell-selective conditions (Fig. 4A) and had downregulated the CSC marker CD61/beta-3 integrin^25^ (Fig. 4B), while the expression of the CSC marker CD29/beta-1 integrin^26^ was not affected (Fig. 4C). Nevertheless, this set of experiments demonstrates that the loss of *Il7*-expressing CAFs impinges on tumor cell stemness *in vivo*, suggesting that these cells form a genuine niche for breast CSC maintenance.

### Oncogenic gene expression marks Il7-positive breast CAFs

**Figure 5.**
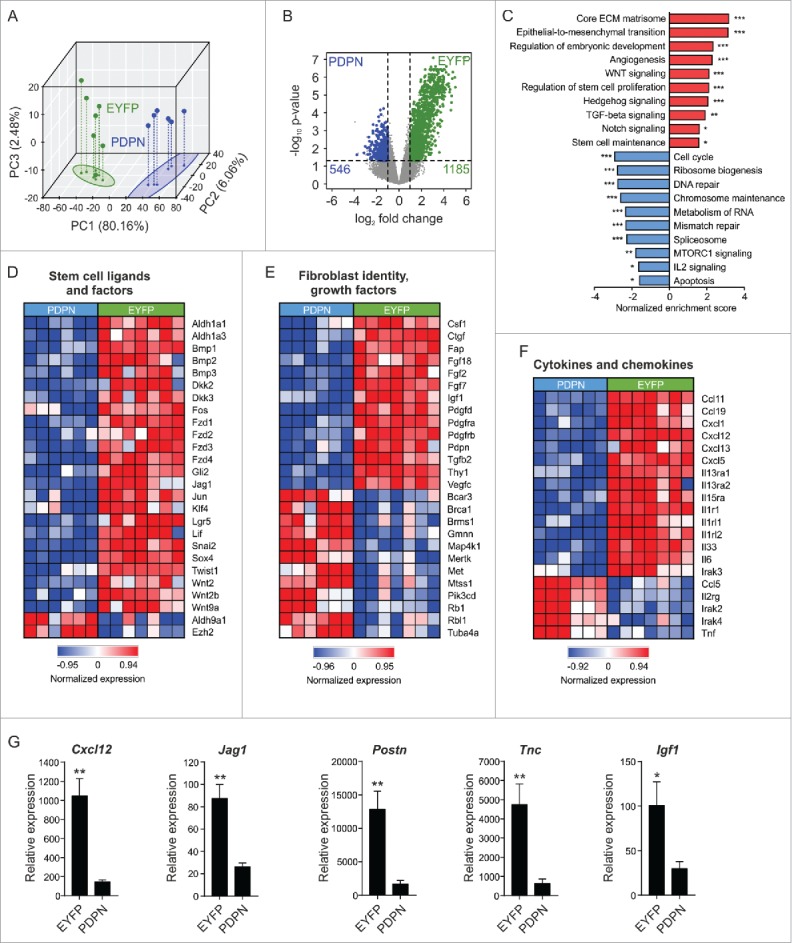
Oncogenic signature and stem cell factor expression by *Il7*-producing fibroblasts. Unilateral E0771 tumors were induced in Il7-EYFP mice and EYFP^+^PDPN^+^ (‘EYFP’) and EYFP^−^PDPN^+^ (‘PDPN’) CAFs were sorted from day 14–16 tumors. Samples were processed and microarray analysis was conducted. (A) 3D PCA of EYFP^+^ (n = 7) and PDPN^+^ (n = 6) breast CAFs. (B) Volcano plot analysis indicating differential expression of 1731 genes. (C) Functional pathway analysis by GSEA. (D-F) Heatmap analysis indicating upregulation of various growth- and stem cell factors as well as cytokines and chemokines by EYFP^+^ CAFs. (G) RT-PCR-based validation of microarray data for the indicated genes (n = 7–8). Statistical testing used: Mann-Whitney U test for panel G (for statistical details on panels A-F see *Microarray analysis*) (*p < 0.05, **p < 0.01, ***p < 0.001).

To better define the mechanisms underlying the tumor-supportive function of *Il7*-expressing CAFs, we compared the gene expression profiles of sorted EYFP^+^PDPN^+^ (designated ‘EYFP’) and EYFP^−^PDPN^+^ (designated ‘PDPN’) CAFs from E0771 tumors growing in Il7-EYFP mice. Bioinformatic analysis revealed that the two cell populations separated well in principal component analysis (PCA) (Fig.5A) and that EYFP^+^ CAFs had up upregulated 1185 genes (Fig.5B). Gene set enrichment analysis (GSEA) showed that the upregulated genes in EYFP^+^ CAFs are components of critical oncogenic pathways associated with cancer progression and poor prognosis (e.g., epithelial-to-mesenchymal transition, angiogenesis) or stemness-related pathways (e.g., WNT, Hedgehog and Notch) (Fig.5C). In contrast, genes associated with functions of fundamental cell integrity, such as chromosome maintenance and DNA repair were downregulated in EYFP^+^ CAFs (Fig.5C). Gene-specific analyses showed upregulation of various extracellular matrix (ECM) components in EYFP^+^ CAFs, indicative of strong fibrogenic and matrix-remodeling potential (Supplementary Fig. 5). Consistently, EYFP^+^ CAFs showed higher expression of principal stem cell ligands and stemness-related genes (Fig.5D), several growth factors (Fig.5E) and a particular set of cancer-related cytokines and chemokines (Fig.5F). Elevated mRNA expression of the microenvironmental stemness regulators *Cxcl12*,^27^^,^^28^ *Jag1*,^29^ *Postn*,^16^ *Tnc*^30^ and *Igf1*^31^ in EYFP^+^ CAFs was validated by RT-PCR (Fig.5G). Moreover, upregulation of *Il33, Fgf2, Tgfb2* and *Vegfc* expression was confirmed and *Il7*, which was not identified as a differentially regulated gene in the microarray, was found to be elevated in EYFP^+^ CAFs (Supplementary Fig. 6). In sum, these data reveal a distinct oncogenic signature of CAFs marked by the Il7-Cre transgene and provide a catalogue of potential candidate targets for stroma-directed BC treatment.

**Figure 6.**
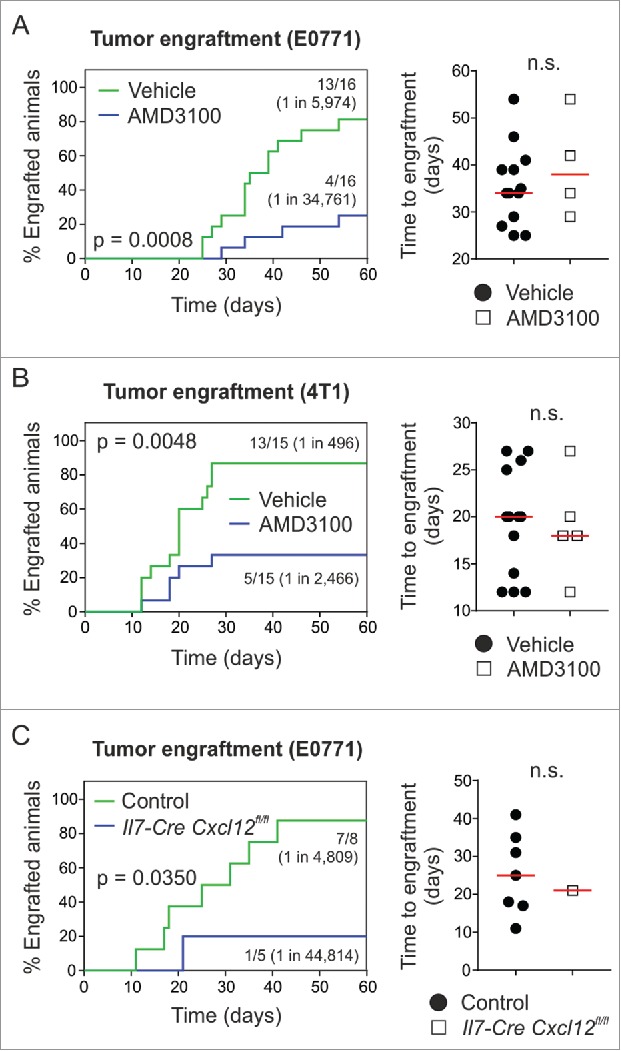
Pharmacological targeting of CXCR4 or conditional ablation of *Cxcl12* depletes breast tumor-initiating potential. (A) 10^4^ E0771 or (B) 10^3^ 4T1 cells were orthotopically grafted in syngeneic wild-type hosts (i.e., C57BL/6 or BALB/c) and animals were treated systemically (i.p.) with 5 mg/kg AMD3100 or vehicle (PBS) on days 0 and 3 post tumor challenge, respectively (n = 15–16). Tumor outgrowth and time to engraftment were monitored. (C) 10^4^ E0771 cells were orthotopically grafted in Il7-Cre *Cxcl12*^fl/fl^ hosts and Cre-negative controls (carrying homozygous *Cxcl12*^fl/fl^ alleles), and tumor outgrowth and time to engraftment were monitored (n = 5–8). Statistical testing used: Log-rank test for Kaplan-Meier curves, and Mann-Whitney U test for the remaining panels.

### The CXCL12/CXCR4 axis constitutes a niche for BC stemness

Of those factors highly upregulated in *Il7*-expressing CAFs, CXCL12 is best known for a role in BC metastasis^32^ and stemness.^27^ We therefore set out to assess the impact of the CXCL12/CXCR4 axis using compounds that block receptor-ligand interaction. Using AMD3100-mediated antagonism of CXCR4, we found that engraftment of E0771 cells was dependent on this particular pathway (1 in 5,974 vehicle vs. 1 in 34,761 AMD3100) (Fig.6A). Likewise, blocking the CXCL12/CXCR4 pathway during engraftment of 4T1 mammary carcinoma cells in BALB/c mice yielded similar results (1 in 496 vehicle vs. 1 in 2,466 AMD3100) (Fig.6B). Interestingly, the time to engraftment (defined as the first day of palpation) was equal between the two groups (Fig. 6A and B), suggesting that early cellular interactions in the tumor cell niche impact the outcome of the seeding process. We next crossed Il7-Cre mice with *Cxcl12*^fl/fl^ mice and found that cell type-specific genetic ablation of *Cxcl12* expression substantially reduced the frequency of tumor-initiating cells (1 in 4,809 control vs. 1 in 44,814 Il7-Cre *Cxcl*12^fl/fl^) without affecting the time to engraftment (Fig.6C). Overall, these data show that interfering with the CXCL12/CXCR4 axis provides a means to therapeutically impact BC stemness and suggest that focused targeting of CAFs that provide the CXCL12-dependent niche could stall metastasis and relapse in BC.

### CXCL12 expression in primary human breast CAFs

**Figure 7.**
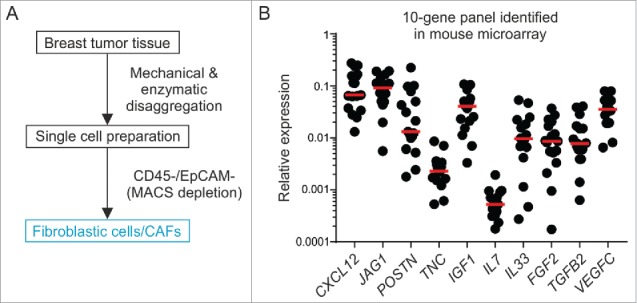
Primary human breast CAFs express stemness-related genes including *CXCL12*. (A) Schematic illustrating the workflow and strategy for isolating CAFs from fresh surgical BC specimens. (B) RT-PCR analysis of enriched breast CAFs for a 10-gene panel identified in the mouse microarray (n = 17).

To establish translational relevance and validate potential stromal cell candidate targets in humans, we isolated fibroblasts from fresh surgical specimens of BC patients undergoing debulking surgery using a double-depletion MACS strategy for CD45 (labeling leukocytes) and EpCAM (labeling epithelial/tumor cells)^33^ (Fig.7A). On average, we were able to recover 6.9 × 10^3^ putative CAFs per mg tumor tissue and achieved routinely a cell purity of >97%. We utilized RT-PCR-based analysis of 10 signature genes identified in the murine *Il7*-expressing breast CAFs to determine whether potential niche factors are expressed in the human cells. All analyzed genes were expressed at detectable levels and showed high variation between patients (e.g., *POSTN, IL33, FGF2*) (Fig.7B). In line with the murine data (Supplementary Fig. 6), expression of *IL7* was found to be low. In contrast, *CXCL12* was one of the most highly expressed genes supporting the notion of the induction of a particular oncogenic gene signature in fibroblasts within the human breast TME. Altogether, these data point towards a phenotypic convergence of murine and human breast CAFs and confirm the expression of potential BC stemness targets in human fibroblastic stromal cells.

## Discussion

In this study, we describe a hitherto unknown population of murine breast tumor-promoting fibroblasts that are characterized by *Il7* promoter activity. These cells surround the tumor mass in two independent models of BC and form network-like structures in areas that putatively harbor CSCs.^34^ *Il7*-expressing breast CAFs were spatially associated with the tumor microvasculature and directly interacted with BC cells, presumably engaging in reciprocal signaling circuits.^35^ *In vivo* ablation of *Il7*-expressing CAFs inhibited tumor growth and depleted the pool of clonogenic (stem-like) cancer cells. We therefore conclude that a defined minor subset of CAFs is crucial for maintaining BC growth and stemness.

*Il7*-expressing breast CAFs showed elevated expression of oncogenic key molecules including growth factors, ECM components, stem cell ligands, cytokines and chemokines. Many of these factors impact on the TME and can govern tumor progression and metastasis through direct (tumor cell-dependent) or indirect (e.g., immune cell-mediated) means. For example, CXCL12 was identified as one of the known factors that regulate BC metastasis^32^^,^^36^ therapy resistance,^37^ activation of estrogen receptors^38^ and breast CSC self-renewal.^27^ Employing orthotopic mammary carcinoma models in a standard readout for the assessment of CSC content,^39^ we uncovered an important role of the CXCL12/CXCR4 axis during the early engraftment phase. Specifically, we found that targeted ablation of *Cxcl12* in Il7-Cre^+^ stromal cells abrogates the tumor-initiating potential of BC cells and that this niche dependence can be therapeutically harnessed through antagonism of CXCR4. The finding that treatment with AMD3100 did not impair the growth of established tumors (data not shown) supports our interpretation that CXCL12 acts on tumor cells that possess stemness rather than the whole tumor cell population, and is consistent with previous reports.^40^^,^^41^ Considering that metastasis is initiated specifically from CSCs,^16^^,^^34^ our data are in line with other recent findings showing that neutralization of CXCR4 inhibits metastasis to lungs and regional lymph nodes in a preclinical model of BC.^32^ Hence, it is possible that early tumor cell-fibroblastic stromal cell interactions dependent on the CXCL12/CXCR4 pathway govern tumor propagation from few malignant cells under both primary and metastatic conditions. It will be important in future studies to elaborate the processes that determine *Cxcl12* induction in a specialized CAF subset during seeding of the tumor niche.

The potent niche activity of CXCL12 is well-known from research on bone marrow and targeted inhibition of CXCL12/CXCR4 signaling is clinically used to mobilize hematopoietic stem cells (HSCs) into circulation. Importantly, Il7-Cre*^+^* cells,^42^ Prx1-Cre*^+^* cells^43^ and *Cspg4/Nestin-Cre^+^* cells^44^ have been identified as cellular source of CXCL12 for HSC maintenance. The findings presented here are in line with these data and suggest overlapping cellular and molecular niche requirements of physiological and malignant stem cells.

BC is a highly heterogeneous disease that requires extensive histological and molecular profiling to provide a basis for individualized treatment decisions. Accordingly, the compelling therapeutic advances of the last 15 years have not benefited all patients, and improving the clinical management of triple-negative disease remains a critical medical need.^45^ From a conceptual viewpoint, the herein presented approach of targeting a particular CAF population holds several advantages over classical (tumor cell-directed) therapies: (i) broad applicability owing to a conserved TME activation pattern among subtypes, (ii) prevention of quick mutational adaptation based on the genetically-stable nature of the targeted cells and (iii) efficacy against various tumor cell subpopulations including those endowed with stem cell properties (niche effect).

It is important to stress that besides CXCL12/CXCR4, our study disclosed further potential targets for breast CSC-directed therapy, such as *Jag1, Postn, Tnc*, and *Il33*. These factors have previously been shown to play a role in the regulation of cancer stemness^16^^,^^29^^,^^30^^,^^46^ and our results show that these potential BC niche-determining factors are expressed in human breast CAFs. Considering that most of the prognostic power of gene expression profiles arises from stromal rather than malignant cells,^47^ the stage is set to further evaluate stromal cell-derived factors in future studies that can be validated in well-designed human cohorts. Overall, this approach will be instrumental to further refine patient stratification and tailor therapies according to precision medicine principles.

## Materials and methods

*Cell lines and culture conditions*. The murine BC cell lines E0771 (C57BL/6)^48^ and 4T1 (BALB/c)^49^ were purchased from CH3 BioSystems and ATCC, respectively. The stable tdT-expressing subline E0771-tdT was generated using lipofection (Invitrogen) with the pLVX-tdTomato-C1 vector (Clontech) and subcloning by limiting dilution. Cells were cultured in RPMI-1640 containing L-glutamine (Sigma-Aldrich), 20 mM HEPES (Life Technologies), 10% (*v/v*) FBS (Sigma-Aldrich), and 100 U/ml penicillin/streptomycin (Lonza). The medium for E0771-tdT cells contained 5 µg/ml puromycin (Life Technologies).

*Mice*. BAC-transgenic Il7-Cre mice^50^ were kindly obtained from Dr. Ellen Richie (MD Anderson). Wild-type (C57 BL/6, BALB/c), *R26R*-EYFP (B6.129 × 1-*Gt(ROSA)26Sor^tm1(EYFP)Cos^*/J) and *R26R*-iDTR (C57 BL/6-*Gt(ROSA)26Sor^tm1(HBEGF)Awai^*/J) mice were purchased from Charles River, and *Cxcl12*^fl/fl^ (B6(FVB)-*Cxcl12^tm1.1Link^*/J) mice were purchased from The Jackson Laboratory. Mice were kept in a specific pathogen-free environment in individually ventilated cages and used in experiments at the age of 6–12 weeks. Sequences of typing primers can be given upon request. Experiments were performed in strict accordance with Swiss federal and cantonal guidelines (‘Tierschutzgesetz’) under the permission numbers SG02/14, SG01/16 and SG09/14 granted by the Veterinary Office of the Canton of St. Gallen.

*Preparation of primary human breast CAFs*. Fresh surgical BC specimens were obtained from the Department of Pathology at the Kantonsspital St. Gallen during July 2016 and July 2017 (n = 17) based on the general consent of patients that permits analysis of tissues without patient‐identifiable data. Samples were mechanically disaggregated and single cell suspensions were generated using gentleMACS technology according to the manufacturer's instructions (Miltenyi Biotec). CAFs were subsequently enriched using MACS-depletion of leukocytes (anti-CD45) and epithelial/tumor cells (anti-EpCAM) (Miltenyi Biotec), and viability of sorted cells was confirmed by Trypan blue exclusion. Enriched CAFs were preserved at −80°C in RNAprotect Cell Reagent (Qiagen) until RNA extraction was performed. The average purity of the recovered CAFs exceeded 97%.

*Syngeneic orthotopic tumor grafts*. E0771, E0771-tdT or 4T1 breast tumors were induced by injecting 5 × 10^5^ cells into the 4th mammary fat pad (MFP) of female mice (total volume 30 µl). Bilateral tumors were induced by injecting tumor cells on both sides (ipsilateral vs. contralateral). Tumor growth was monitored with calipers and tumor size was calculated using the formula tumor volume = ½ (length × width^2^).^51^ For *in vivo* ablation of *Il7*-expressing CAFs, 1 ng of DT was intratumorally administered on days 6, 8 and 10 post tumor challenge, respectively (total volume 10 µl). Upon harvesting, tumors were either preserved as (i) MagNA Lyser-mashed tissues at −80°C in TRI Reagent (Sigma-Aldrich), (ii) fixed in paraformaldehyde (Merck), or (iii) freshly dissociated using mechanical disruption and enzymatic digestion with a dispase/collagenase/DNase cocktail (Roche and Sigma-Aldrich). To determine the significance of the CXCL12/CXCR4 axis for BC tumorigenic potential, pre-titrated limiting doses of E0771 (10^4^) and 4T1 (10^3^) cells were transplanted into syngeneic immunocompetent hosts (C57BL/6 or BALB/c). Animals were treated systemically (i.p. injection) with 5 mg/kg of the CXCR4 antagonist AMD3100 (Sigma-Aldrich) on days 0 and 3 post tumor challenge, respectively, and monitored for tumor growth at least twice a week. Mice not showing tumor outgrowth by day 60 were considered non-engrafted. To investigate the specific contribution of *Il7*-positive CAF-derived *Cxcl12* to BC tumorigenic potential, 10^4^ E0771 cells were grafted into Il7-Cre *Cxcl12*^fl/fl^ mice, and animals were monitored for tumor outgrowth for up to 60 days as well. The frequency of tumorigenic cells was calculated using L-Calc Software version 1.1 (STEMCELL Technologies).

*Flow cytometry*. Flow cytometric analyses were done on a two-laser FACSCanto II or a four-laser LSRFortessa, both operated by FACSDiva version 8.0.1 software (BD Biosciences). Staining of surface antigens was performed at a density of 10^6^ cells/100 µl in PBS containing 1% (*v/v*) FBS (Sigma-Aldrich) for 25 min at 4°C. Stained cells were washed in PBS, chilled on ice, and analyzed within an hour. EYFP expression by Il7-Cre^+^ cells and tdT expression by tumor cells was detected using a 530/30 filter behind a 505 long-pass and a 586/15 filter behind a 550 long-pass, respectively. Dead cells were excluded based on forward/side scatter characteristics and/or 7-AAD positivity (BioLegend), and doublets and aggregates were discriminated by comparing the different signals of forward scatter. Data were finally analyzed using FlowJo version 10 (Tree Star). Flow cytometry antibodies are listed in Supplementary Table 1.

*Cell sorting by FACS*. Flow cytometric sorting was performed on a two-laser S3 Cell Sorter operated by ProSort version 1.4 software (Bio-Rad). For microarray analysis, EYFP^+^ and EYFP^−^PDPN^+^ breast CAFs were sorted from day 14–16 E0771 tumors. Cells were sorted into tubes prefilled with RNAprotect Cell Reagent (Qiagen) and stored at −80°C. In another set of experiments, E0771-tdT cells were sorted from ablated and non-ablated day 14 tumors. These cells were live-purified and immediately sub-cloned to assess their clonogenic potential. Samples were stained and processed exactly as described above and the average purity of all recovered cell fractions exceeded 95%.

*Single cell clonogenicity*. As surrogate readout for tumoral stemness, *ex vivo*-purified cancer cells were counted and diluted to the limiting density of 5 cells/ml medium. 150 µl of this suspension (corresponding to 0.75 cells) were seeded into 96-well ultra-low attachment plates (Corning) and cultured for 2 weeks to allow clonal outgrowth. The number of wells harboring viable (tdT-expressing) clones was counted under a fluorescence microscope (Leica), and the percentage clonogenicity was determined.

*Histology*. Tumors and tdLNs (inguinal) were harvested and fixed overnight in 4% (*w/v*) paraformaldehyde (draining activity of the ipsilateral inguinal lymph node was demonstrated using *in vivo* tracking of intratumorally administered 40 kDa FITC-dextran; data not shown). Tissues were washed in PBS containing 1% (*v/v*) Triton-X 100 (Sigma-Aldrich) and 2% (*v/v*) FBS. Samples were embedded in 4% (*w/v*) low-meting agarose (Peqlab) and cut into 40 µm sections using a VT-1200 Vibratome (Leica Biosystems). Unspecific binding was reduced through blocking with 1 mg/ml anti-FcγR antibodies (BD Biosciences), and sections were incubated overnight with the following antibodies: anti-EYFP (Clontech), anti-PDPN (eBioscience), anti-αSMA (Sigma-Aldrich), anti-CD31 (BioLegend), and anti-Ki-67 (eBioscience). Primary antibodies were detected with the following secondary reagents: Alexa488-conjugated anti-rabbit-IgG, DyLight549-conjugated anti-Syrian hamster-IgG, and Alexa647-conjugated streptavidin (Jackson ImmunoResearch). tdT expression by tumor cells did not require antibody-mediated signal amplification for visualization. Nuclei were stained with DAPI (Life Technologies) and sections were mounted onto glass slides using specialized fluorescence medium (Dako). Samples were analyzed on an LSM-710 confocal microscope operated by ZEN 2010 software (Zeiss), and images were processed in Imaris version 7.7.1 (Bitplane).

*PCR-based expression analysis*. RT-PCR was performed on a LightCycler 480 II instrument operated by LightCycler 480 SW 1.5 software (Roche). Amplification was done using the LightCycler FastStart DNA Master SYBR Green I kit (Roche), and either TBP (mouse) or GAPDH (human) was used as housekeeping gene. Reactions were performed in duplicate and relative gene expression was calculated using the ΔΔCt method. Primer assays are provided in Supplementary Table 2.

*RNA isolation, cDNA generation and microarray pre-processing*. RNA was isolated from sorted cells using the RNeasy Mini Kit (Qiagen), and from mashed tissue using the Direct-zol RNA MiniPrep Kit (Zymo Research). Contaminating DNA was eliminated through on-column DNase digestion (Zymo Research). To generate cDNA for RT-PCR analysis, the High-Capacity cDNA Reverse Transcription Kit was employed (Applied Biosystems). To generate cDNA for microarray analysis, the Ovation Pico WTA System V2 was used followed by fragmentation and biotinylation using the Encore Biotin Module (NuGEN). Microarrays were run on the GeneChip Mouse Gene 2.1 ST platform (Affymetrix) at the Kompetenzzentrum für Fluoreszente Bioanalytik (KFB) of the University of Regensburg. Prior to hybridization, cDNA products were quality-controlled using gel separation and the best 8 pairs of samples (of altogether 11) were chosen for analysis.

*Microarray analysis*. Gene expression analysis was performed using R and Bioconductor.^52^ Raw gene expression data were normalized using the robust multi-array average (RMA) algorithm^53^ in the *oligo* package.^54^ Quality control was performed on all samples according to Affymetrix quality control workflow and identified outlier samples were removed from subsequent analyses. Differential gene expression was determined using the empirical Bayes moderated t-test with a Benjamini-Hochberg multiple comparison correction^55^ implemented in the *limma* package.^56^ FC was calculated as the difference in RMA-normalized log_2_-transformed pairwise group-mean gene expression values. |FC| >2 and adjusted p-value <0.05 selection thresholds were used to determine differentially expressed genes. A total of 1731 differentially expressed genes were found between EYFP^+^ and EYFP^−^PDPN^+^ breast CAFs. PCA was performed for the 1731 differentially expressed genes in order to determine sample phenotype segregation for the first three principal components. Volcano plots were generated for log_2_ FC values and log_10_ adjusted p-values for comparison between EYFP^+^ and EYFP^−^PDPN^+^ cells. Heatmaps were visualized using log_2_-transformed, row-mean centered and row-normalized gene expression values. GSEA^57^ was performed for all genes from Il7-EYFP and PDPN-expressing fibroblasts on the following gene sets: H hallmark, C2 canonical pathways, C5 Gene Ontology biological processes, C6 oncogenic signatures and C7 immunologic signatures. Normalized enrichment scores were calculated for 1000 permutations, with a minimum gene set size of 15 genes and a maximum of 500 genes. False discovery rate-adjusted p-values were calculated using the empirical gene set-based permutation test. Adjusted p-values <0.05 were considered statistically significant.

*Statistics*. GraphPad Prism 7 (GraphPad Software) was used for all statistical analyses. Tests were two-sided and differences with a p-value <0.05 were considered significant. Unless otherwise stated, experiments were performed on at least two, but typically three or more, separate occasions, and data were pooled. Data are shown as mean ± SEM, or as individual data points with the median value indicated.

## Supplementary Material

2017ONCOIMM0797R-s02.docx
