# Supplementary material for: Interleukin 7-expressing fibroblasts promote breast cancer growth through sustenance of tumor cell stemness
Source: Oncoimmunology. 2018 Jan 3;7(4):e1414129. doi: 10.1080/2162402X.2017.1414129 (PMC5889213; doi:10.1080/2162402X.2017.1414129)
Supplement: 2017ONCOIMM0797R-s02.docx [file koni-07-04-1414129-s001.docx]

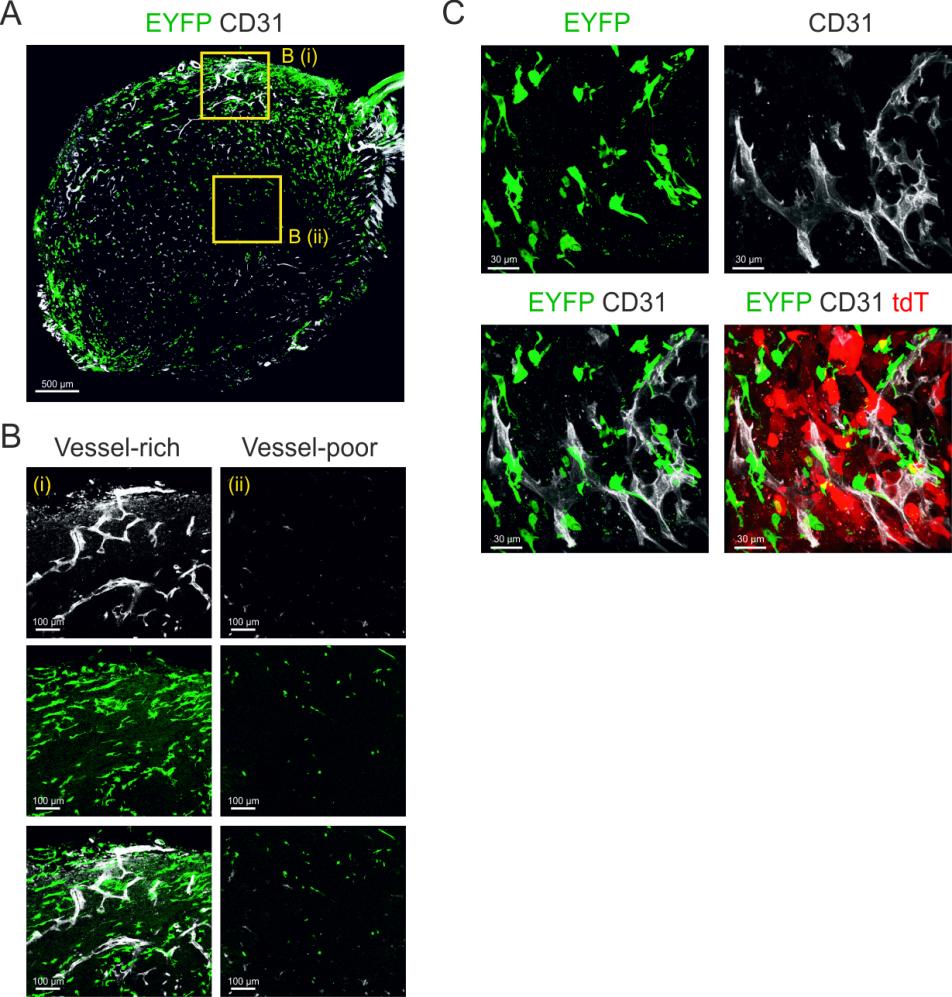


**Supplementary Figure 1. Spatial association of EYFP^+^ CAFs and CD31^+^ blood vessels.** 5×10^5^ E0771 (A) or 5×10^5^ E0771-tdT (C) cells were grafted in the MFP of *Il7-EYFP* mice and tumors were harvested at day 14. Tissue sections were stained for EYFP and CD31 and analyzed by confocal microscopy. (B) Boxed area close-ups of (A) depicting respective vessel-rich and –poor regions. Data are representative examples of at least three independently performed experiments (n > 3).


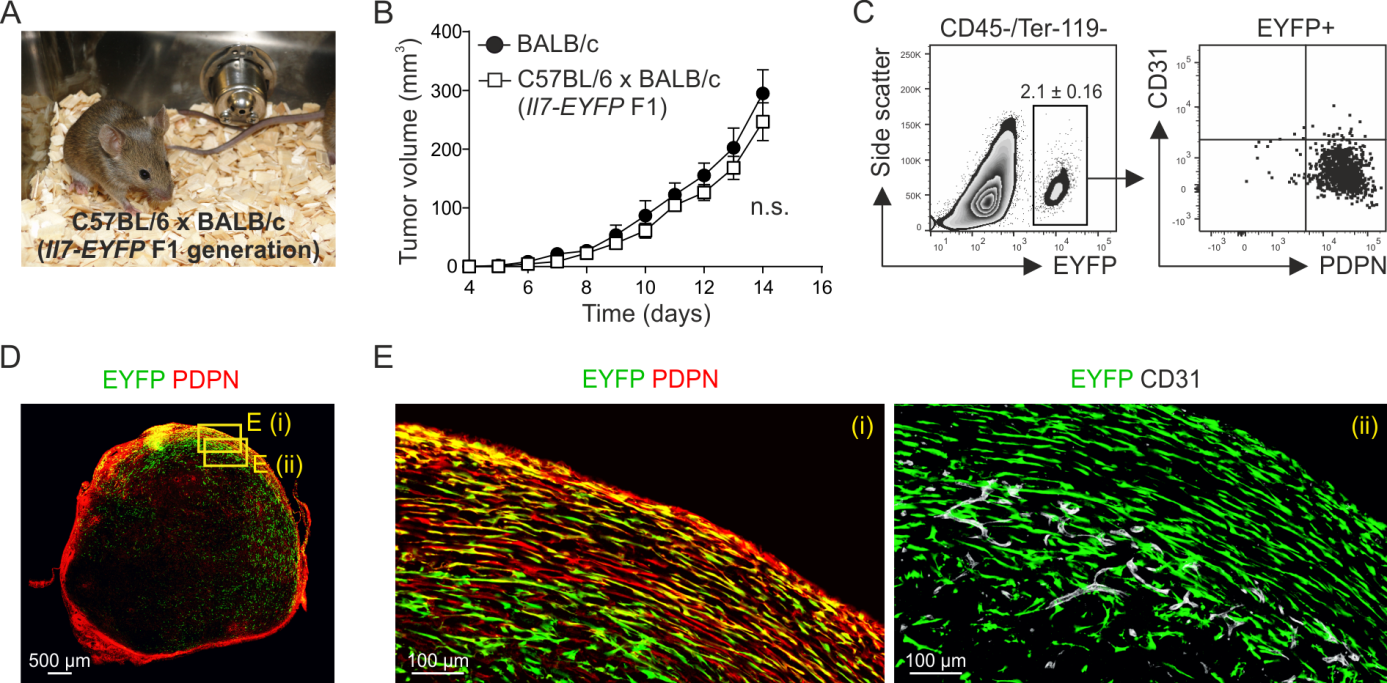


**Supplementary Figure 2. Analysis of EYFP^+^ CAFs in 4T1 tumors.** *Il7-EYFP* (C57BL/6) mice were bred to BALB/c mice to generate F1 offsprings with an immune-compatible environment for 4T1 breast tumor take (50/50 mixed genetic background). Tumors were induced in these animals by orthotopic grafting of 5×10^5^ 4T1 cells. (A) Picture of an F1 intercross. (B) Comparative 4T1 tumor growth in F1 vs. BALB/c mice (n = 5). (C) Flow cytometric characterization of EYFP^+^ cells in 4T1 tumors of F1 mice (n = 4). (D+E) Confocal microscopic analysis of EYFP^+^ fibroblasts in 4T1 tumors of F1 mice (n = 4). Statistical testing used: Repeated measures ANOVA for panel B. Data are from one experiment, respectively.


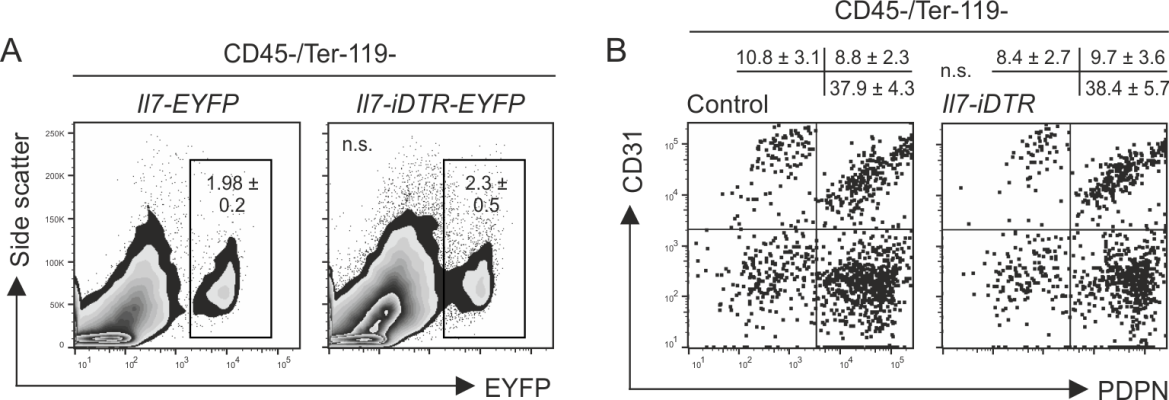


**Supplementary Figure 3. Impact of toxin-mediated ablation of *Il7*-expressing CAFs on the infrastructure of the tdLN.** E0771 cells were orthotopically grafted in *Il7-iDTR/EYFP* mice and transgene-expressing CAFs were ablated by intratumoral administration of DT (1 ng on days 6, 8 and 10). (A) Flow cytometric analysis of EYFP^+^ cells in the tdLN at day 14 (n = 3). (B) Flow cytometric analysis of various stromal cell populations defined by PDPN and CD31 expression in the tdLN (control mice had an iDTR-positive genetic background but lacked Cre expression) (n =5). Statistical testing used: Student’s t-test for panels A and B.

**
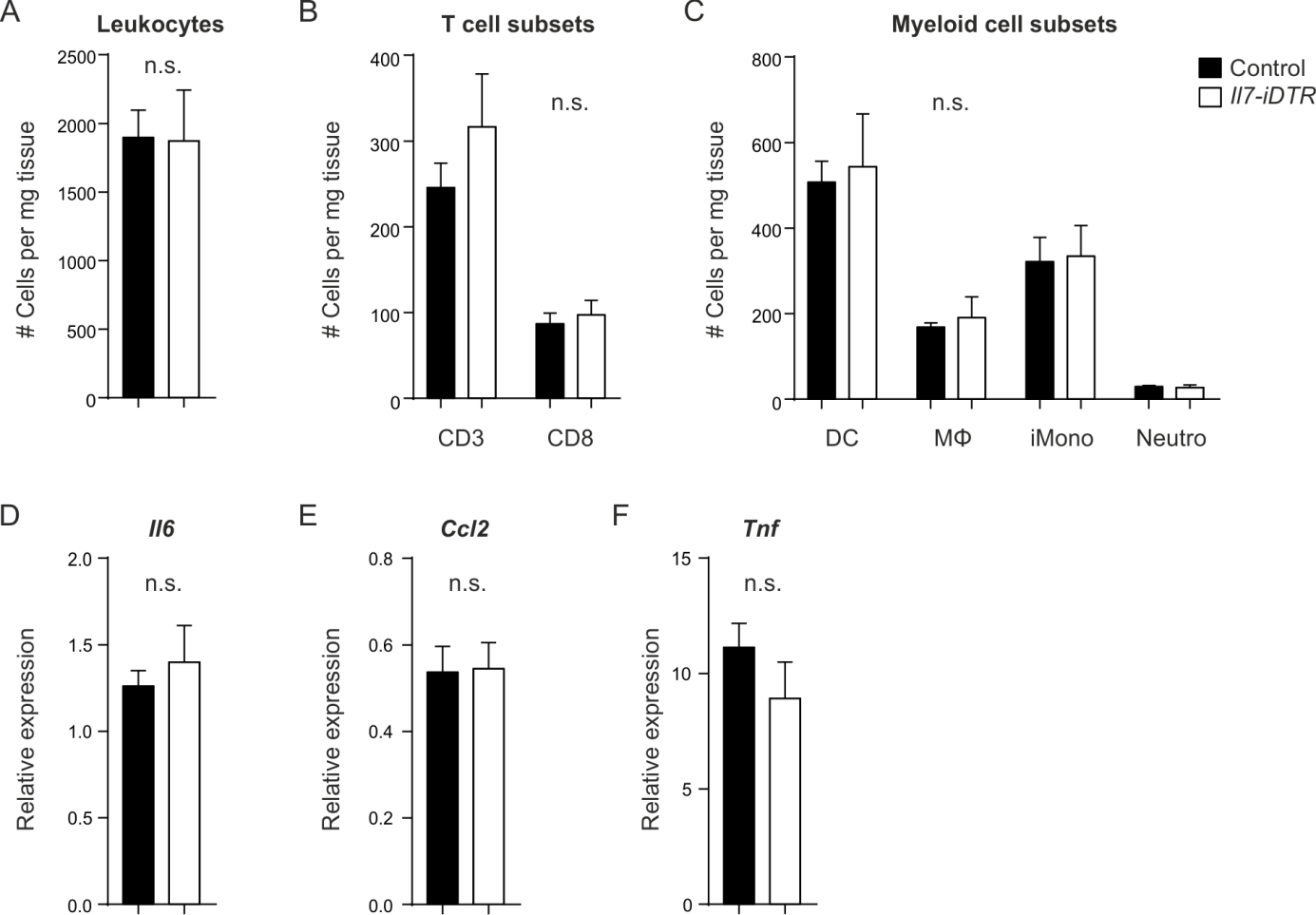
**

**Supplementary Figure 4. Impact of toxin-mediated ablation of *Il7*-expressing fibroblasts on breast tumor growth.** E0771 cells were orthotopically grafted in *Il7-iDTR/EYFP* mice and transgene-expressing CAFs were ablated by intratumoral administration of DT (1 ng on days 6, 8 and 10). (A-C) Flow cytometric quantification of various immune cell populations on day 11, expressed as absolute numbers of cells per mg tumor tissue (n = 7). Myeloid cell definition: Dendritic cell (DC), *CD11c+/F4/80-*; Macrophage (MΦ), *CD11b+/F4/80+*; inflammatory monocyte (iMono), *CD11b+/F4/80-/Ly6C+*, Neutrophil (Neutro), *CD11b+/F4/80-/Ly6Cdim/Ly6G+*. (D-F) RT-PCR for the indicated inflammatory mediators performed on bulk tumor tissue on day 11 (n = 7). Statistical testing used: Student’s t-test for panels A-C, and Mann-Whitney U test for panels D-F.


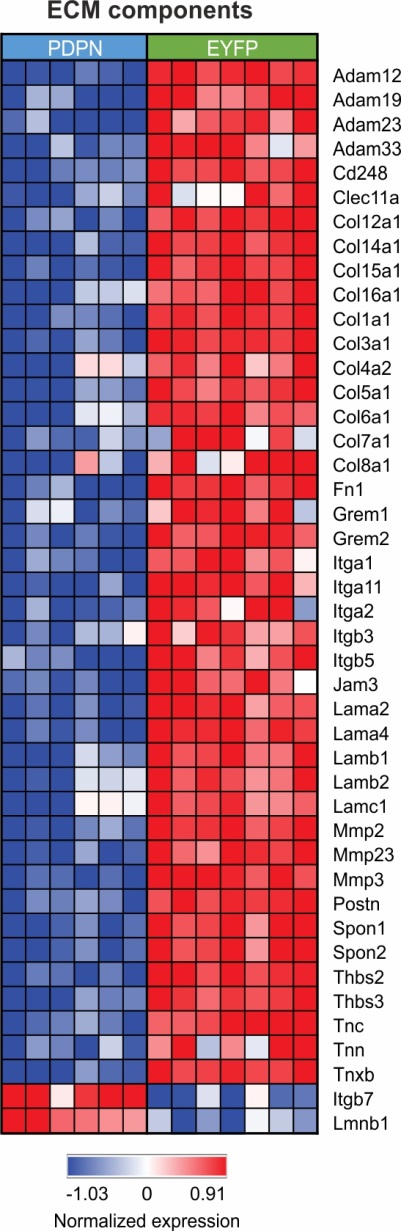


**Supplementary Figure 5. Gene expression signatures associated with fibrosis and ECM-remodeling potential.** Heatmap analysis of the **Figure 5** dataset for ECM-related genes (for statistical details see *Microarray analysis*).


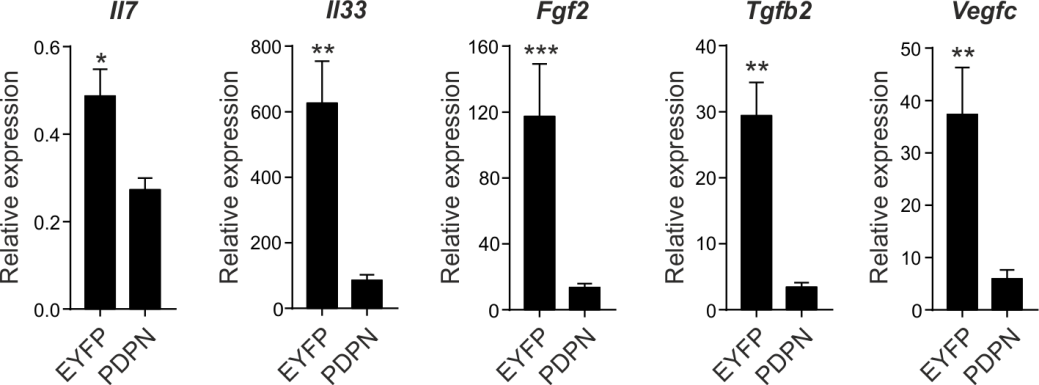


**Supplementary Figure 6. Validation of microarray-based gene expression analysis.** RT-PCR-based analysis of the samples shown in **Figure 5G** for the indicated genes (n = 7-8). Statistical testing used: Mann-Whitney U test (*p<0.05, **p<0.01, ***p<0.001).

**Supplementary Table 1. List of flow cytometry antibodies used in this study.**

| **Antigen** | **Clone** | **Supplier** |
| --- | --- | --- |
| **Mouse** |  |  |
| CD3 | 145-2C11 | BioLegend |
| CD4 | RM4-5 | BioLegend |
| CD8 | 53-6.7 | BioLegend |
| CD11b | M1/70 | BioLegend |
| CD11c | N418 | eBioscience |
| CD29 (beta-1 integrin) | HMβ1-1 | BioLegend |
| CD31 | MEC13.3 | BioLegend |
| CD44 | IM7 | BioLegend |
| CD45 | 30-F11 | BioLegend |
| CD54 (ICAM-1) | 3E2 | BD Biosciences |
| CD61 (beta-3 integrin) | 2C9.G2 (HMβ3-1) | Miltenyi Biotec |
| CD62L | MEL-14 | BioLegend |
| CD90 | 30-H12 | BioLegend |
| CD105 | MJ7/18 | BD Biosciences |
| CD106 (VCAM-1) | 429 (MVCAM.A) | BioLegend |
| CD140a (PDGFRα) | APA5 | eBioscience |
| CD140b (PDGFRβ) | APB5 | eBioscience |
| CD274 (PD-L1) | 10F.9G2 | BioLegend |
| Anti-sheep IgG | Polyclonal | Abcam |
| FAPα | Polyclonal sheep | R&D Systems |
| F4/80 | BM8 | eBioscience |
| I-A/I-E (MHC class II) | M5/114.15.2 | BioLegend |
| Ly-6C | HK1.4 | BioLegend |
| Ly-6G | 1A8 | BD Biosciences |
| PDPN | 8.1.1 | BioLegend |
| Sca-1 | D7 | BioLegend |
| Streptavidin | ——— | BD Biosciences |
| Ter-119/erythroid | TER-119 | BioLegend |
| **Human** |  |  |
| CD31 | WM59 | eBioscience |
| CD45 | HI30 | eBioscience |
| EpCAM | EBA-1 | BD Biosciences |

**Supplementary Table 2. List of RT-PCR primers used in this study.**

| **Gene** | **Primer Assay/Sequence** | **Supplier** |
| --- | --- | --- |
| **Mouse** |  |  |
| *Ccl2* | QT00167832 | Qiagen |
| *Cxcl12* | QT00161112 | Qiagen |
| *Fgf2* | QT00128135 | Qiagen |
| *Igf1* | QT00154469 | Qiagen |
| *Il6* | QT00098875 | Qiagen |
| *Il7* | fwd GTGCCACATTAAAGACAAAGAAG  rev GTTCATTATTCGGGCAATTACTATC | Microsynth |
| *Il33* | QT00135170 | Qiagen |
| *Jag1* | QT00115703 | Qiagen |
| *Postn* | QT00150759 | Qiagen |
| *Tbp* | fwd CCTTCACCAATGACTCCT  rev CAAGTTTACAGCCAAGAT | Microsynth  Microsynth |
| *Tgfb2* | QT00106806 | Qiagen |
| *Tnc* | QT00106176 | Qiagen |
| *Tnf* | QT00104006 | Qiagen |
| *Vegfc* | QT00104027 | Qiagen |
| **Human** |  |  |
| *CXCL12* | QT00087591 | Qiagen |
| *FGF2* | QT00047579 | Qiagen |
| *GAPDH* | QT00079247 | Qiagen |
| *IGF1* | QT00029785 | Qiagen |
| *IL7* | QT02411227 | Qiagen |
| *IL33* | QT00041559 | Qiagen |
| *JAG1* | QT00031948 | Qiagen |
| *POSTN* | QT00023800 | Qiagen |
| *TGFB2* | QT00025718 | Qiagen |
| *TNC* | QT00024409 | Qiagen |
| *VEGFC* | QT00061579 | Qiagen |
